# Space changes after premature loss of mandibular second primary molars: a systematic review and meta-analysis

**DOI:** 10.1186/s12903-026-10044-8

**Published:** 2026-10-30

**Authors:** Ahmed Kamal El-Motayam, Yousra Mohamed Abdel Rehim, Mais Medhat Sadek, Mai Hamdy Ragab, Reham ELnemr, Nour Wahba

**Affiliations:** 1 Department of Pediatric Dentistry and Dental Public Health, Faculty of Dentistry, Cairo University, 11 Al Saraya street, Manial, Cairo, Egypt; 2 Department of Pediatric Dentistry, Faculty of Dentistry, Ahram Canadian University, Cairo, Egypt; 3 University of Sharjah, P.O. Box 82828, Sharjah, United Arab Emirates; 4 Ain Shams University, El- Khalifa El-Mamoun Street, Abbasya, Cairo, Egypt; 5 Department of Endodontics, Faculty of Dentistry, Suez Canal University, 4.5 km ring road, Ismailia, Egypt; 6 Department of Pediatric Dentistry and Dental Public Health, Faculty of Dentistry, Suez Canal University, 4.5 km ring road, Ismailia, Egypt; 7 Department of Pediatric Dentistry, Faculty of Dentistry, Ain Shams University, El-Khalifa El-Mamoun Street, Abbasya, Cairo, Egypt

**Keywords:** Primary molars, Space loss, Space maintainer, Systematic review, Meta-analysis, Premature extraction

## Abstract

**Background:**

Deciduous dentition plays a crucial role in mastication, phonetics, and maintaining arch space for permanent successors. While several systematic reviews have quantified spatial changes following first primary molar loss, specific evidence synthesis for second primary molars is lacking. This systematic review and meta-analysis aimed to quantify space loss following premature extraction of second primary molars and evaluate the clinical necessity of space maintenance.

**Methods:**

Five electronic databases (PubMed, Web of Science, LILACS, EMBASE, and Cochrane libraries) were searched without date restrictions (last search 03/06/2025). Inclusion criteria included randomized, quasi-randomized, and non-randomized clinical trials involving children aged 4–9 years with second primary molar indicated for extraction. Two independent investigators assessed studies for inclusion, evaluated risk of bias using ROBINS-I tool, and extracted data. Random-effects models with 95% confidence intervals calculated mean differences as effect measures.

**Results:**

Four studies were included in qualitative synthesis, with three studies involving 38 children included in meta-analysis. Meta-analysis demonstrated mean mandibular space loss of 0.80 mm (95% CI: -1.00 to -0.60; *p* < 0.0001) at 3 months, 1.10 mm (95% CI: -1.24 to -0.96; *p* < 0.0001) at 6 months, and 1.24 mm (95% CI: -1.48 to -1.02; *p* < 0.0001) at 9–10 months post-extraction. Arch hemi-perimeter decreased by 1.06 mm (95% CI: -1.26 to -0.31; *p* = 0.001) at 6 months.

**Conclusions:**

Current limited short‑term evidence suggests that premature mandibular second primary molar loss in children with erupted first permanent molars results in mean space loss of approximately 1.24 mm over 9‑10 months. Whether this short‑term space loss translates into long‑term malocclusion or orthodontic treatment need remains unknown. Therefore, clinical decisions regarding space maintenance should remain individualized and based on arch length discrepancy, occlusal development, crowding, molar relationship, and long‑term orthodontic risk. The current evidence is insufficient to support routine space maintainer use. Maxillary arch evidence is too limited to support any conclusion.

**Protocol registration:**

PROSPERO CRD42025640491.

**Supplementary Information:**

The online version contains supplementary material available at https://doi.org/10.1186/s12903-026-10044-8.

## Introduction

Deciduous dentition plays a crucial role in mastication, phonetics, and maintaining arch space for normal eruption of permanent successors [1]. Premature loss of primary molars consequently presents a significant clinical challenge, primarily due to the risk of space loss within the dental arch, which can disrupt harmonious occlusal development [2, 3].

Space loss occurs predominantly through mesial drift of first permanent molars and is most rapid within the first six months after extraction [4–6]. The resulting malocclusion may manifest as crowding, altered molar relationships, or impaction of succedaneous premolars [7, 8]. To mitigate these effects, space maintainer placement following premature extraction has become standard clinical protocol aimed at preserving arch length [9].

However, this intervention is not without drawbacks. Space maintainers are associated with increased risk of caries and periodontal inflammation, require strict patient compliance, and can fail or decementation occurs, adding financial burden on families [10–12]. This raises a critical question: do the benefits of space maintenance consistently outweigh potential drawbacks for every primary tooth?

This question is particularly pertinent for second primary molars (Es). The eruption of second premolars into their space is a key event in the transition to permanent dentition. However, the precise amount and clinical significance of space loss following premature extraction remain poorly quantified and are subjects of ongoing debate [13].

While several systematic reviews have quantified spatial changes following first primary molar loss [14–16], specific evidence synthesis for second primary molars is lacking. The magnitude of space loss, definitive need for space maintainers, and the balance of risks and benefits for this specific situation remain unclear .

This systematic review and meta-analysis aimed to: (1) quantify the amount and pattern of space loss following premature extraction of Es, and (2) evaluate the clinical necessity of space maintenance based on available evidence.

## Methods

### Protocol and registration

The protocol was registered on the National Institute of Health Research Database (www.crd.york.ac.uk/prospero, Protocol: CRD42025640491).

### Eligibility criteria

Inclusion criteria: Prospective longitudinal observational studies with a split‑mouth design (i.e., comparing the extraction side to the contralateral intact side in the same patient). Randomized and quasi‑randomized clinical trials were also considered if they contained an untreated control arm that provided observational data on natural space changes following extraction. Participants were children aged 4–9 years with a second primary molar indicated for extraction. Studies were required to report quantitative changes in extraction space (E‑space) and/or arch hemi‑perimeter. Exclusion criteria: Literature reviews, case reports, letters to the editor, and cross‑sectional studies.

### Information sources and search strategy

We searched five electronic databases (last search 03/06/2025; no date restrictions): PubMed, Web of Science, LILACS, EMBASE, and Cochrane libraries. Search terms included combinations of: ‘premature or early’, ‘deciduous molars’, ‘primary molars’, ‘temporary molars’, ‘baby molars’, ‘loss or extraction’, ‘space loss’, and ‘migration and drift’, using Boolean operators OR and AND. Reference lists of included studies were also reviewed. To minimize publication bias, we additionally screened grey literature sources, including ProQuest Dissertations & Theses, OpenGrey, and conference proceedings from the International Association for Dental Research (IADR) and the European Orthodontic Society (EOS) for the period 2000–2025.

### Study selection and data extraction

Two independent investigators (A.K.E. and N.W.) assessed studies for inclusion, evaluated risk of bias, and extracted data. Disagreements were resolved through discussion and consultation with a third author (M.M.). We developed a standardized data extraction form recording: author, year, study design, sample size, participant age, tooth extracted, follow-up duration, and outcome data.

### Risk of bias assessment

Study quality was assessed using the ROBINS-I tool, evaluating seven domains: confounding, participant selection, intervention classification, deviations from intended interventions, missing data, outcome measurement, and selective reporting. For randomized controlled trials in which only a single arm (e.g., an untreated control group) contributes data to the synthesis, and no randomized between-group comparison is analysed, the resulting data may be considered observational in nature. In such cases, risk of bias was assessed using the ROBINS-I tool, which is designed for evaluating non-randomized estimates of intervention effects. We acknowledge that this approach involves methodological judgement, and the rationale has been reported transparently to allow readers to appraise it accordingly.

### Statistical analysis

We assessed clinical heterogeneity by examining variations in participant characteristics, study design, and measurement techniques. Statistical heterogeneity was evaluated using forest plots, confidence intervals, I² statistics, and chi-square tests. Statistical heterogeneity was considered present at *p* < 0.05, with I² > 50% interpreted as moderate to high heterogeneity. Random-effects models with 95% confidence intervals were used to calculate mean differences (MD) as effect measures. This model was selected over a fixed-effect model because it provides a more conservative estimate when dealing with a limited number of studies and accounts for potential heterogeneity arising from variations in study populations or unmeasured methodological differences. All analyses were performed using R programming software.

## Results

### Study selection and characteristics

**Fig. 1:**
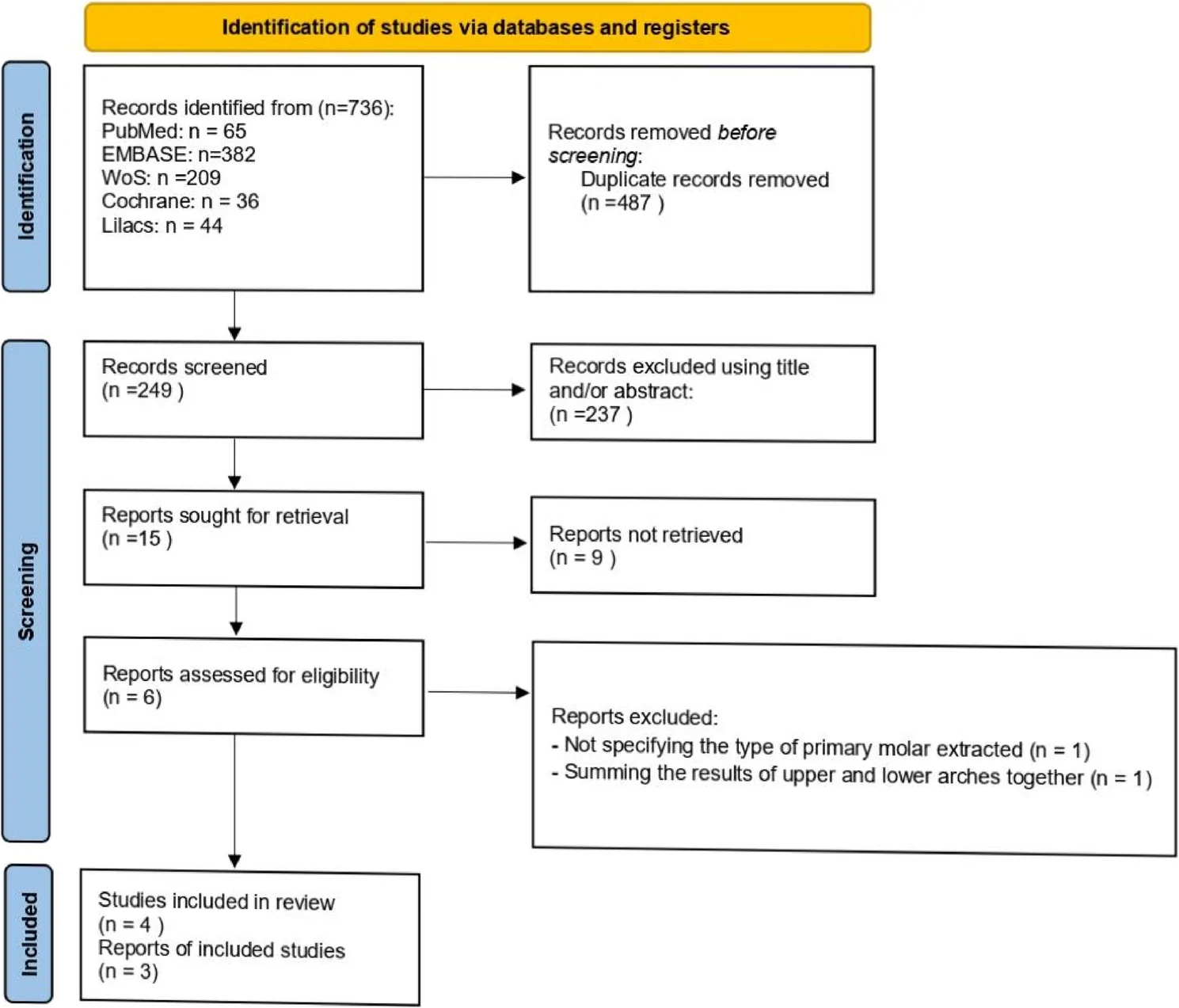
PRISMA flow diagram showing the study selection process

The study selection process is summarized in Fig. 1. The systematic search yielded 736 records initially. After removing 487 duplicates, 249 records were screened by title and abstract. Following exclusions, 15 reports were sought for retrieval, with nine reports unavailable [17–25]. Six full-text articles were assessed for eligibility, with two excluded: one for not specifying primary molar type [26], another for combining upper and lower arch results [27]. Screening of grey literature sources identified no additional eligible studies.

Four studies were included in qualitative synthesis: three longitudinal observational studies with split-mouth design [28–30] and one two-arm randomized clinical trial [31]. The characteristics of the included studies are summarized in Table 1.The Bindayel et al. study [29] was excluded from quantitative meta-analysis due to its short three-week follow-up period. Meta-analysis was performed on the remaining three studies involving 38 children.

**Table 1:** Characteristics of included studies

Author, year	Study design	Country	Sample size (*n*)	Age (years)	Arch	Extracted tooth	Follow‑up	Main finding on space loss
Macena et al., 2011 [28]	Longitudinal observational, split‑mouth	Brazil	55 (22 mandibular second molar included in meta‑analysis)	6‑9	Maxilla & mandible	Second primary molar (subset)	3, 6, 10 months	Significant space loss only for second molars; greatest loss in first 3 months
Bindayel, 2019 [29]	Longitudinal observational, split‑mouth	Saudi Arabia	32	7.5‑10.7 (mean 9 ± 1.5)	Maxilla & mandible	Second primary molar	3 weeks	Statistically significant space loss (0.43 mm); excluded from meta‑analysis due to short follow‑up
Kundra et al., 2024 [30]	Longitudinal observational, split‑mouth	India	20 (6 mandibular second molar included in meta‑analysis)	6‑9 (mean 6.95 ± 0.83)	Maxilla & mandible	Second primary molar (subset)	9 months	Significant reduction in arch width, length, hemi‑perimeter, and extraction space
de Souza et al., 2024 [31]	Randomised clinical trial (only control arm used)	Brazil	26 (control arm *n* = 10)	6‑9 (mean 7.45 ± 0.92)	Mandible	First or second primary molar	3, 6 months	Control group had greater space loss at 3 months; no difference between groups at 6 months

### Risk of bias assessment

**Fig. 2:**
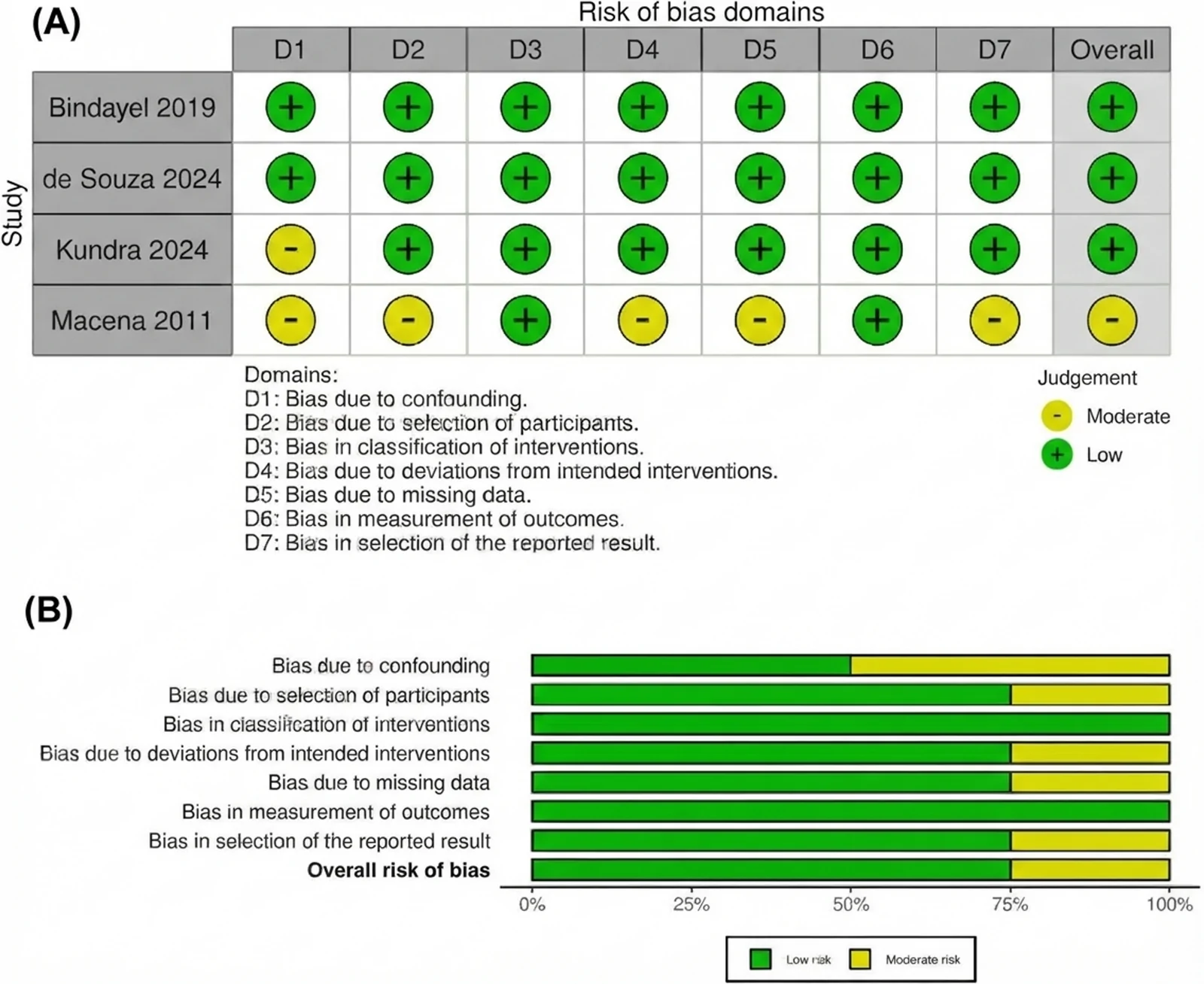
Risk of Bias evaluation: traffic presentation (**A**) and summary for each domain (**B**)

All included studies were assessed for risk of bias, and results were summarized in Fig. 2. The quality assessment using the ROBINS-I tool revealed varying levels of risk of bias across domains.

Among the included studies, de Souza et al. [31] was originally designed as a randomized controlled trial. However, only the untreated control arm (which received no space maintainer and was followed prospectively) contributed data to the quantitative synthesis, as the intervention arm involved active treatment not relevant to the present research question. As a result, the data included in the meta-analysis represent observational follow-up without a randomized between-group comparison. This control arm therefore provides data that are functionally comparable to those of a prospective cohort study, reflecting natural space changes following extraction in the absence of intervention. Accordingly, risk of bias was assessed using the ROBINS-I tool, and the study was judged to have an overall low risk of bias within the context of this methodological approach.

Two additional observational studies [29, 30] also demonstrated low overall risk of bias, indicating high methodological rigor and reliability. The study of Macena et al. [28] was assessed as having a moderate overall risk of bias, primarily due to moderate risks identified in confounding, selection bias, and missing data.

### Meta-analysis results

#### Mandibular space loss following premature extraction

**Fig. 3:**
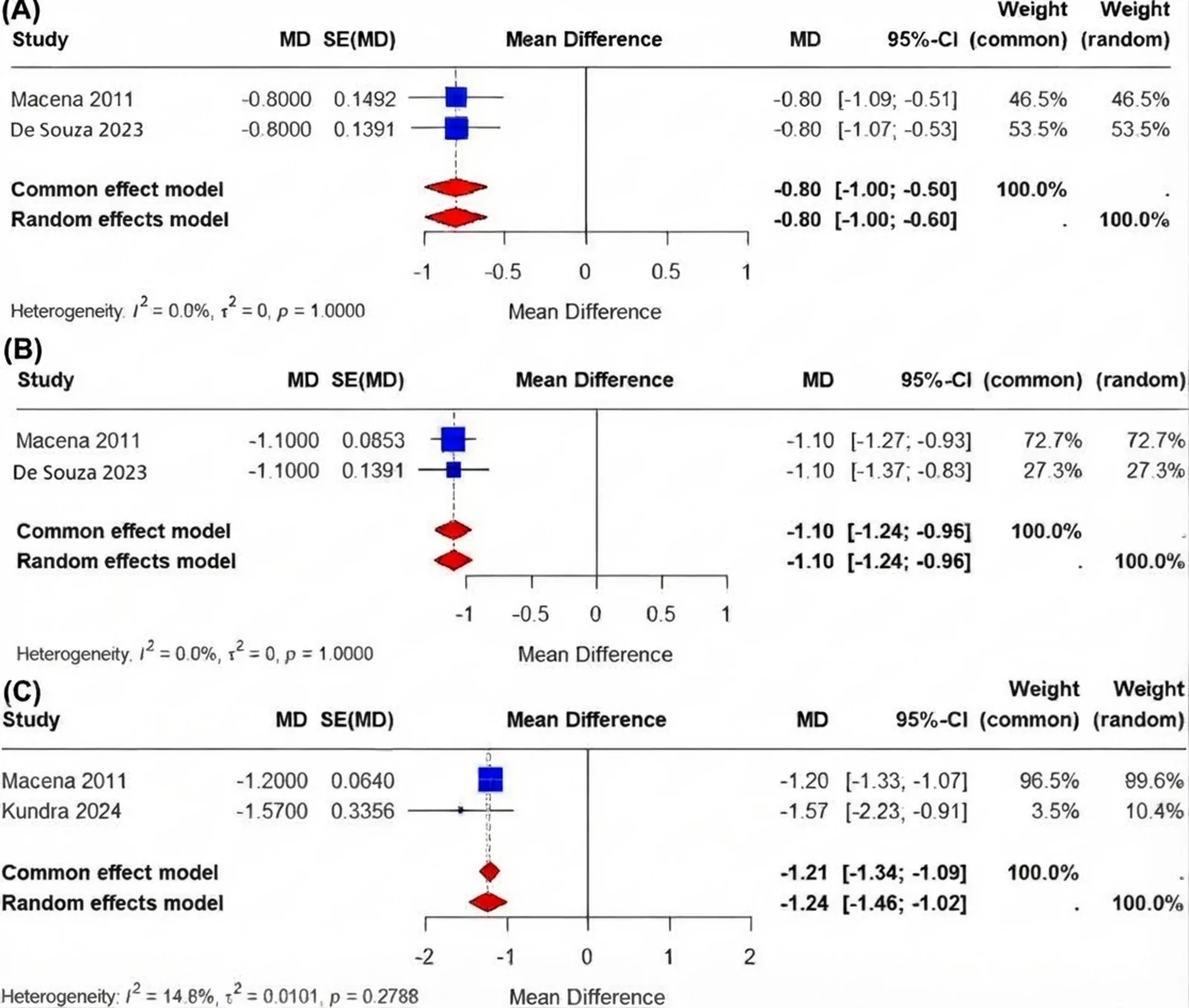
Forest plots for mandibular space loss: (**A**) after 3 months (**B**) after 6 months (**C**) after 9-10 months

Space loss in mandibular second primary molar extraction sites was assessed in all three studies included in quantitative synthesis ( Fig. 3). At 3 months, meta-analysis demonstrated mean space loss of 0.80 mm (95% CI: -1.00 to -0.60; *p* < 0.0001) with no statistical heterogeneity (I² = 0%; chi-square *p* = 0.497). At 6 months, space loss was 1.10 mm (95% CI: -1.24 to -0.96; *p* < 0.0001) with no heterogeneity (I² = 0%). At 9–10 months, space loss reached 1.24 mm (95% CI: -1.48 to -1.02; *p* < 0.0001) with minimal heterogeneity (I² = 14%).

#### Arch hemi-perimeter changes

**Fig. 4:**
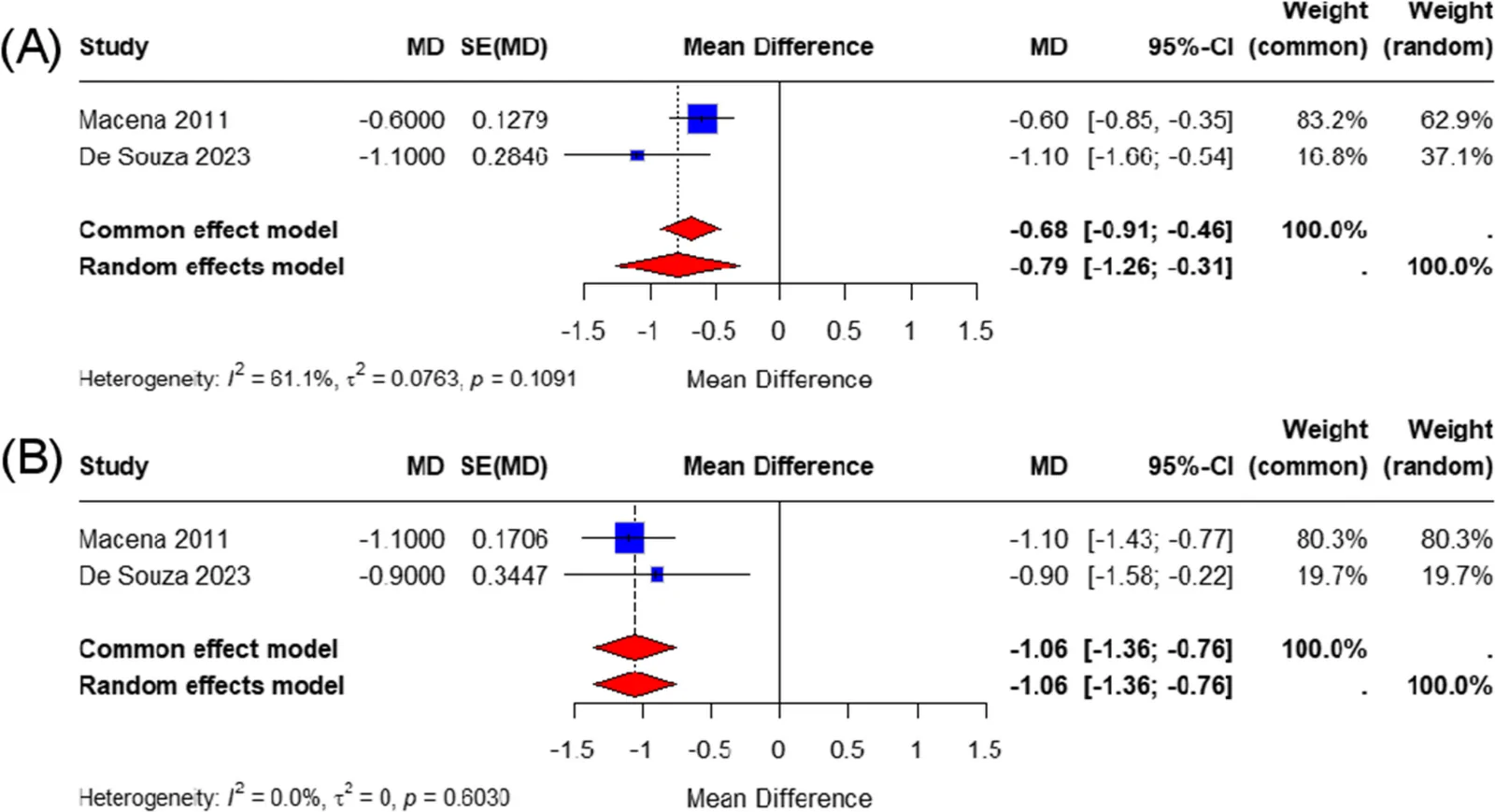
Forest plots for arch hemi-perimeter changes: (**A**) after 3 months (**B**) after 6 months

Changes in mandibular arch hemi-perimeter were evaluated in two studies (Fig. 4). At 3 months, pooled analysis showed mean decrease of 0.79 mm (95% CI: -1.26 to -0.31; *p* = 0.001) with moderate heterogeneity (I² = 61.1%; chi-square *p* = 0.08). At 6 months, reduction was 1.06 mm (95% CI: -1.26 to -0.31; *p* = 0.001) with no heterogeneity (I² = 0%).

#### Maxillary arch changes

Data on maxillary arch changes were limited and reported narratively. Macena et al. [28] found a mean space loss of 1.2 ± 0.8 mm at 3 months, with minimal subsequent change (*n* = 13). Kundra et al. [29] reported on two maxillary cases, noting a decrease in E-space from 9.45 ± 0.35 mm to 8.35 ± 0.92 mm post-extraction. Macena et al. also reported minimal change in maxillary arch hemi-perimeter over the follow-up period (*P* = 0.440). No conclusions can be drawn due to the very limited data.

## Discussion

### Summary of evidence

This systematic and meta-analysis quantified spatial changes following premature mandibular second primary molar extraction. While the evidence is based on a limited number of studies, results from three studies [28, 30, 31] show consistent patterns of space loss, with mean space loss progressing from 0.80 mm at 3 months to 1.24 mm at 9–10 months post-extraction. Concurrent arch hemi-perimeter decrease of 1.06 mm at 6 months indicates space closure occurs primarily through mesial movement of first permanent molars All quantitative findings apply only to the mandibular arch; maxillary arch data were too sparse for meta-analysis, and no conclusions can be drawn.

### Clinical interpretation

It is important to note that these findings describe only short-term dimensional changes (up to 10 months after extraction). Whether this degree of space loss leads to clinically significant malocclusion, permanent tooth impaction, or increased need for orthodontic treatment in the long term is not addressed by the available studies and remains unknown. Therefore the following considerations are not intended as formal clinical guidelines but as preliminary observations to inform individualised decision-making.

The 1.24 mm space loss observed in this review may represent an initial manifestation of late mesial shift, the normal developmental migration of first permanent molars into the available E-space, which stands for the difference between the mesiodistal widths of the second primary molar and its successor premolar [32]. This movement is essential for establishing proper Class I molar relationships [33]. However, the clinical significance of this physiological space utilization depends critically on the individual patient’s arch length discrepancy (ALD)—the difference between available arch perimeter and the mesiodistal widths of permanent teeth to be accommodated [34].

In children with no crowding or dental spacing (ALD ≥ 0 mm) [35], the observed 1.24 mm loss is substantially below the 2.5 mm E-space threshold and represents normal physiological drift. In such patients, one could hypothesize that placing a space maintainer might interfere with the natural occlusal adjustment required for physiologic molar relationship establishment, converting a developing Class I relationship into Class II and increasing the likelihood of second permanent molar impaction [36]. The long-term consequences of short-term space loss remain unknown.

In children with mild crowding (negative ALD up to 3 mm), every millimeter of arch perimeter becomes clinically meaningful. The additional 1.24 mm of arch perimeter loss from extraction may be sufficient to push the patient into the moderate crowding range, crossing the 3 mm threshold. Close monitoring may be warranted, and space maintenance could be considered on an individual basis.

In children with moderate crowding (negative ALD of 3–6 mm), the 1.24 mm extraction-related loss could represent an additional burden on an already compromised arch perimeter. For such patients, space maintenance might be considered, but the decision should be based on individual clinical judgment, as evidence of long-term benefit is lacking.

In children with severe crowding (negative ALD exceeding 6 mm), the pre-existing arch length deficit is likely the primary determinant of occlusal outcome. These patients will probably need comprehensive orthodontic planning, which may include extraction of permanent teeth, irrespective of space maintenance after primary molar loss.

### Comparison with Existing Literature

The pattern of space loss observed in this review—characterized by rapid initial closure followed by a plateau—is consistent with the established natural history following premature loss of primary molars. Our quantitative synthesis for mandibular second primary molars shows progression from 0.80 mm at 3 months to 1.24 mm at 9–10 months. This pattern parallels that reported for first primary molars, where the most significant space loss also occurs in the early months post-extraction [16].

The immediate onset of this process is highlighted by Bindayel [29], who documented statistically significant space loss of 0.43 mm within just three weeks of extraction. While data from this very early timepoint are limited for first primary molars, available evidence consistently indicates that the fastest rate of closure occurs within the first few months post-extraction [16]. This fundamental biological pattern of rapid mesial drift appears consistent across primary molar types, though the clinical implications may differ based on the specific tooth lost and timing relative to permanent successor eruption.

### Limitations

This review is limited by the small number of eligible studies (*n* = 3) and the consequent small pooled sample size (*n* = 38 children). This constrains statistical power, generalizability, and the ability to conduct meaningful subgroup analyses. The assessment of publication bias was also not feasible. To address the limited data, we employed conservative random-effects models.

The small number of studies has important implications for the precision of our estimates. While the confidence intervals for mandibular space loss were relatively narrow (e.g., 95% CI: − 1.48 to − 1.02 at 9–10 months), suggesting acceptable precision for these specific results, the overall evidence base remains fragile. Precision is influenced by both sample size and effect consistency, and the low observed heterogeneity (I² = 0–14%) suggests reproducible effects across studies—a factor that partially compensates for the small sample. However, with only 2–3 studies per meta-analysis, the statistical power to detect heterogeneity is inherently low; thus, I² values should be interpreted with caution, as the absence of detected heterogeneity does not confirm homogeneity [37].

Furthermore, because so few studies contribute to these analyses, precision estimates could be substantially altered by the addition of a single new study. Consequently, while our point estimates (e.g., 1.24 mm space loss) appear stable, confidence in them as accurate reflections of the true underlying effect is necessarily limited. The evidence should therefore be considered preliminary, and clinicians should interpret the findings with appropriate caution.

An additional methodological consideration relates to the three split‑mouth studies included [28–30]. Although these studies appropriately used paired analyses at the individual study level, the within‑patient correlation coefficients were not reported and could not be incorporated into the meta‑analysis – a common limitation when synthesizing split‑mouth designs [37]. The absence of this correlation adjustment may have led to underestimation of variance and potential overprecision of the pooled estimates; consequently, confidence intervals should be interpreted with caution.

The application of ROBINS-I to the untreated control arm of the de Souza et al. [31] randomized trial represents a non-standard methodological approach. Although this arm was treated as observational in nature, this classification involves a degree of judgement. Accordingly, the risk of bias assessment for this study and conclusions drawn from its data should be interpreted with appropriate caution, in light of its original randomized design. This is acknowledged as a methodological limitation of the present review.

Our findings are primarily specific to the mandibular arch. Data on maxillary arch changes were severely limited and too heterogeneous for quantitative synthesis. The available narrative data from included studies suggested variable space loss [28, 30], but the very small number of maxillary cases precludes any definitive conclusions. This critical gap in the literature means our results and clinical recommendations cannot be reliably extended to the premature loss of maxillary second primary molars.

Inconsistent reporting in primary studies presents additional limitations. Measurement techniques lacked sufficient detail for reproducibility assessment, and several patient‑ and tooth‑related factors that may influence space loss were not consistently reported. These include pre‑existing crowding or spacing, molar relationship, the eruption stage of the permanent successor, the presence or absence of intercuspation of the first permanent molars, the extent of caries and interproximal wear of adjacent teeth, and underlying craniofacial growth patterns [38, 39]. The omission of these variables limits our ability to assess their impact on the results and the generalisability of the findings across different clinical presentations. Consequently, the observed space loss estimates represent average effects across heterogeneous populations, and the influence of individual baseline characteristics on the degree of space loss remains largely unknown.

The absence of non-extraction control groups represents a methodological consideration. While pre-extraction baselines provide robust within-subject comparisons, control groups would better account for normal developmental changes. However, evidence suggests that arch dimensions in the intact primary dentition remain stable over short periods. This is supported by Zhao et al.‘s meta-analysis, which found no significant changes in arch width, length, or perimeter in untreated dentitions over 6–24 months [16].

An additional limitation is the inability to retrieve nine potentially eligible reports identified through our search [17–25]. These studies, published between 1967 and 1982, lacked accessible contact information for the authors, and full texts could not be obtained despite extensive efforts through inter-library loans and institutional repositories. Their exclusion may introduce a risk of selection bias, as unavailable studies could differ in the magnitude or direction of reported space changes, potentially influencing the pooled estimates.

Perhaps the most significant limitation is the absence of long-term occlusal outcome data following second primary molar extraction. While our review quantifies immediate spatial consequences, whether these changes translate to clinically meaningful malocclusion remains unexplored. Interestingly, Lin et al. [40] documented increased total arch dimensions years after early primary first molar extraction, suggesting remarkable dentoalveolar adaptation capacity over time. Although this study examined first rather than second primary molars and had methodological limitations, it highlights that long-term occlusal development is a dynamic, multifactorial process that cannot be predicted solely from short-term space changes.

Despite these limitations, this review provides the first quantitative synthesis of space loss after premature mandibular second primary molar extraction. However, given the small number of studies and modest sample size, all conclusions should be considered preliminary until confirmed by larger, high‑quality, long‑term investigations.

## Conclusion

Within the limitations of the available evidence, premature mandibular second primary molar loss in children with erupted first permanent molars results in mean space loss of approximately 1.24 mm over 9‑10 months. This loss may represent an initial phase of physiological late mesial shift, being substantially less than the 2.5 mm E‑space required for normal occlusal development. All quantitative findings apply only to the mandibular arch; the available maxillary data are too limited to support any conclusion or recommendation.

The available evidence describes only short‑term space changes (up to 10 months after extraction). Whether these changes translate into long‑term malocclusion, impaction, or future orthodontic treatment need is unknown. Therefore, clinical decisions regarding space maintenance should remain individualised and based on arch length discrepancy, occlusal development, crowding, molar relationship, and long‑term orthodontic risk. The current evidence is insufficient to support routine space maintainer use.

These conclusions should be considered preliminary given the small evidence base. Significant knowledge gaps remain, and further high‑quality, long‑term research is essential for developing definitive clinical guidelines.

## Supplementary Information

Supplementary Material 1: PRISMA checklist

Supplementary Material 2: Search strategy

Supplementary Material 3: Supplementary Table S1: Detailed numerical outcomes per study (mean ± SD at each time point)

## Data Availability

The datasets analyzed in this study are available from the corresponding author upon reasonable request.

## Abbreviations

ROBINS-I: Risk of Bias in Non-randomized Studies of Interventions
