# Supplementary material for: Space changes after premature loss of mandibular second primary molars: a systematic review and meta-analysis
Source: BMC Oral Health. 2026 Oct 30;26:2149. doi: 10.1186/s12903-026-10044-8 (PMC13721352; doi:10.1186/s12903-026-10044-8)
Supplement: Supplementary file 1 [file 12903_2026_10044_MOESM1_ESM.docx]

**The PRISMA checklist of this systematic review and meta-analysis**

| **Section and Topic** | **Item #** | **Checklist item** | **Location where item is reported** |
| --- | --- | --- | --- |
| **TITLE** | | |  |
| Title | 1 | Identify the report as a systematic review. | **Title Page: "a systematic review and meta-analysis."** |
| **ABSTRACT** | | |  |
| Abstract | 2 | See the PRISMA 2020 for Abstracts checklist.  Provide a structured summary including, as applicable: background; objectives; data sources; study eligibility criteria participants, and interventions; study appraisal and synthesis methods; results; limitations; conclusions and implications of key findings, systematic review registration number. | **Abstract: Sections: Background, Objectives, Search Methods, Selection Criteria, Data Collection and Analysis, Results, Limitations, Conclusions.** |
| **INTRODUCTION** | | |  |
| Rationale | 3 | Describe the rationale for the review in the context of existing knowledge. | **Introduction:** Final paragraph, explaining the gap for second primary molars. |
| Objectives | 4 | Provide an explicit statement of the objective(s) or question(s) the review addresses. | **Objectives Section:** "the aim of this study was to evaluate the space changes... and assess the need for a space maintainer." |
| **METHODS** | | |  |
| Eligibility criteria | 5 | Specify the inclusion and exclusion criteria for the review and how studies were grouped for the syntheses. | **Materials and Methods -> Eligibility criteria:** Listed as points 1-5. |
| Information sources | 6 | Specify all databases, registers, websites, organisations, reference lists and other sources searched or consulted to identify studies. Specify the date when each source was last searched or consulted. | **Materials and Methods -> Information sources...:** "four electronic databases...", "last updated 03/06/2025” |
| Search strategy | 7 | Present the full search strategies for all databases, registers and websites, including any filters and limits used. | **Materials and Methods -> Information sources...:** Keywords and Boolean operators listed. (Fully detailed in Appendix 1). |
| Selection process | 8 | Specify the methods used to decide whether a study met the inclusion criteria of the review, including how many reviewers screened each record and each report retrieved, whether they worked independently, and if applicable, details of automation tools used in the process. | **Materials and Methods -> Information sources...:** "Two independent investigators... assessed studies for inclusion..." |
| Data collection process | 9 | Specify the methods used to collect data from reports, including how many reviewers collected data from each report, whether they worked independently, any processes for obtaining or confirming data from study investigators, and if applicable, details of automation tools used in the process. | **Materials and Methods -> Data items and collection:** "We developed a data extraction form... Two independent investigators... extracted data." |
| Data items | 10a | List and define all outcomes for which data were sought. Specify whether all results that were compatible with each outcome domain in each study were sought (e.g. for all measures, time points, analyses), and if not, the methods used to decide which results to collect. | **Materials and Methods -> Eligibility criteria (item 5):** "The changes in extraction space (E-space) and changes in arch hemi-perimeter." |
|  | 10b | List and define all other variables for which data were sought (e.g. participant and intervention characteristics, funding sources). Describe any assumptions made about any missing or unclear information. | **Materials and Methods -> Data items and collection:** Lists extracted data: author, year, design, sample size, age, etc. |
| Study risk of bias assessment | 11 | Specify the methods used to assess risk of bias in the included studies, including details of the tool(s) used, how many reviewers assessed each study and whether they worked independently, and if applicable, details of automation tools used in the process. | **Materials and Methods -> Risk of bias...:** "assessed using the ROBINS-I tool... by two independent investigators." |
| Effect measures | 12 | Specify for each outcome the effect measure(s) (e.g. risk ratio, mean difference) used in the synthesis or presentation of results. | **Materials and Methods -> Statistical Analysis...:** "mean difference (MD) as the effect measure." |
| Synthesis methods | 13a | Describe the processes used to decide which studies were eligible for each synthesis (e.g. tabulating the study intervention characteristics and comparing against the planned groups for each synthesis (item #5)). | **Results -> Study selection...:** Describes exclusion of studies that did not meet criteria (e.g., Bindayel excluded from meta-analysis). |
|  | 13b | Describe any methods required to prepare the data for presentation or synthesis, such as handling of missing summary statistics, or data conversions. | **Results -> Meta-analysis:** Describes use of mean difference and 95% CI. |
|  | 13c | Describe any methods used to tabulate or visually display results of individual studies and syntheses. | **Table 1, Figure 1, 2, 3, 4.**. |
|  | 13d | Describe any methods used to synthesize results and provide a rationale for the choice(s). If meta-analysis was performed, describe the model(s), method(s) to identify the presence and extent of statistical heterogeneity, and software package(s) used. | **Materials and Methods** -> Statistical Analysis...: "random-effects model...", "I² statistic and chi-square test." |
|  | 13e | Describe any methods used to explore possible causes of heterogeneity among study results (e.g. subgroup analysis, meta-regression). | Results -> Meta-analysis: Reports I² values for each analysis. |
|  | 13f | Describe any sensitivity analyses conducted to assess robustness of the synthesized results. | **Materials and Methods** -> Additional analyses: "pre-planned sensitivity analyses were conducted." |
| Reporting bias assessment | 14 | Describe any methods used to assess risk of bias due to missing results in a synthesis (arising from reporting biases). | The **Materials and Methods ->** Risk of bias across studies: "funnel plots would be generated..." |
| Certainty assessment | 15 | Describe any methods used to assess certainty (or confidence) in the body of evidence for an outcome. | **Results -> Quality of evidence assessment:** "assessed using GRADE criteria (Table 2)." |
| **RESULTS** | | |  |
| Study selection | 16a | Describe the results of the search and selection process, from the number of records identified in the search to the number of studies included in the review, ideally using a flow diagram. | **Results -> Study selection...:** "initially yielded 736 records..." and **Figure 1: PRISMA flow diagram.** |
|  | 16b | Cite studies that might appear to meet the inclusion criteria, but which were excluded, and explain why they were excluded. | **Results -> Study selection...:** Lists studies not retrieved [22-30] and two excluded after full-text assessment ^31, 32^. |
| Study characteristics | 17 | Cite each included study and present its characteristics. | Table 1: Characteristics of included studies. |
| Risk of bias in studies | 18 | Present assessments of risk of bias for each included study. | Results -> Risk of bias within studies and Figure 2. |
| Results of individual studies | 19 | For all outcomes, present, for each study: (a) summary statistics for each group (where appropriate) and (b) an effect estimate and its precision (e.g. confidence/credible interval), ideally using structured tables or plots. | **Table 1** provides detailed outcome data for each study. |
| Results of syntheses | 20a | For each synthesis, briefly summarise the characteristics and risk of bias among contributing studies. | **Results -> Study selection...: and Risk of bias within studies.** |
|  | 20b | Present results of all statistical syntheses conducted. If meta-analysis was done, present for each the summary estimate and its precision (e.g. confidence/credible interval) and measures of statistical heterogeneity. If comparing groups, describe the direction of the effect. | **Results -> Meta-analysis:** MD and 95% CI for space loss and hemi-perimeter at 3,6,9-10 months. **Figure 3 & 4.**. |
|  | 20c | Present results of all investigations of possible causes of heterogeneity among study results. | **Results -> Meta-analysis:** Reports I² values (e.g., I²=0%, I²=61.1%). |
|  | 20d | Present results of all sensitivity analyses conducted to assess the robustness of the synthesized results. | Not applicable |
| Reporting biases | 21 | Present assessments of risk of bias due to missing results (arising from reporting biases) for each synthesis assessed. | **Results -> Risk of bias across studies...:** "Assessment of publication bias was not undertaken..." |
| Certainty of evidence | 22 | Present assessments of certainty (or confidence) in the body of evidence for each outcome assessed. | **Results -> Quality of evidence assessment and Table 2: GRADE summary of findings.** |
| **DISCUSSION** | | |  |
| Discussion | 23a | Provide a general interpretation of the results in the context of other evidence. | **Discussion ->** Summary of Evidence, Interpretation of results. |
|  | 23b | Discuss any limitations of the evidence included in the review. | **Discussion -> Limitations.** |
|  | 23c | Discuss any limitations of the review processes used. | **Discussion ->** Limitations. (e.g., small number of studies, reporting variability). |
|  | 23d | Discuss implications of the results for practice, policy, and future research. | **Discussion -> Implications for Practice and Research.** |
| **OTHER INFORMATION** | | |  |
| Registration and protocol | 24a | Provide registration information for the review, including register name and registration number, or state that the review was not registered. | **Materials and Methods -> Protocol and registration:** PROSPERO CRD42025640491. |
|  | 24b | Indicate where the review protocol can be accessed, or state that a protocol was not prepared. | **Materials and Methods -> Protocol and registration:** [www.crd.york.ac.uk/prospero](http://www.crd.york.ac.uk/prospero). |
|  | 24c | Describe and explain any amendments to information provided at registration or in the protocol. | The protocol is described in the Methods and Registration (CRD42025640491). |
| Support | 25 | Describe sources of financial or non-financial support for the review, and the role of the funders or sponsors in the review. | **Funding**: self funded |
| Competing interests | 26 | Declare any competing interests of review authors. | The authors declare that they have no competing interests. |
| Availability of data, code and other materials | 27 | Report which of the following are publicly available and where they can be found: template data collection forms; data extracted from included studies; data used for all analyses; analytic code; any other materials used in the review. | The datasets used and/or analyzed during the current study are available from the corresponding author on reasonable request. |
