# Supplementary material for: Space changes after premature loss of mandibular second primary molars: a systematic review and meta-analysis
Source: BMC Oral Health. 2026 Oct 30;26:2149. doi: 10.1186/s12903-026-10044-8 (PMC13721352; doi:10.1186/s12903-026-10044-8)
Supplement: Supplementary file 2 [file 12903_2026_10044_MOESM2_ESM.docx]

**Search strategy**

1- PubMed , Chocrane library and Lilacs

| #1 | primary AND molar* |
| --- | --- |
| #2 | deciduous AND molar* |
| #3 | temporary AND molar* |
| #4 | baby AND molar* |
| #5 | #1 OR #2 OR #3 OR #4 |
| #6 | premature* extract* |
| #7 | premature* los* |
| #8 | early los* |
| #9 | early extract* |
| #10 | #6 OR #7 OR #8 OR #9 |
| #11 | space los* |
| #12 | spatial change* |
| #13 | space change* |
| #14 | migrat* |
| #15 | drift |
| #16 | #11 OR #12 OR #13 OR #14 OR #15 |
| #17 | #5 AND #10 AND #16 |

2- Web of Science

| #1 | TS= primary NEAR/2 molar* |
| --- | --- |
| #2 | TS= deciduous NEAR/2 molar* |
| #3 | TS= temporary NEAR/2 molar* |
| #4 | TS= baby NEAR/2 molar* |
| #5 | #1 OR #2 OR #3 OR #4 |
| #6 | TS= premature* extract* |
| #7 | TS= premature* los* |
| #8 | TS= early los* |
| #9 | TS= early extract* |
| #10 | #6 OR #7 OR #8 OR #9 |
| #11 | TS= space los* |
| #12 | TS= spatial change* |
| #13 | TS= space change* |
| #14 | TS= migrat* |
| #15 | TS= drift |
| #16 | #11 OR #12 OR #13 OR #14 OR #15 |
| #17 | #5 AND #10 AND #16 |

3.EMBASE

| #1 | (primary adj2 molar*).ti,ab,kw. |
| --- | --- |
| #2 | (deciduous adj2 molar*).ti,ab,kw. |
| #3 | (temporary adj2 molar*).ti,ab,kw. |
| #4 | (baby adj2 molar*).ti,ab,kw. |
| #5 | primary tooth/exp |
| #6 | #1 OR #2 OR #3 OR #4 OR #5 |
| #7 | (premature* adj2 extract*).ti,ab,kw. |
| #8 | (premature* adj2 los*).ti,ab,kw. |
| #9 | (early adj2 los*).ti,ab,kw. |
| #10 | (early adj2 extract*).ti,ab,kw. |
| #11 | tooth extraction/exp |
| #12 | #7 OR #8 OR #9 OR #10 OR #11 |
| #13 | (space adj2 los*).ti,ab,kw. |
| #14 | (spatial adj2 change*).ti,ab,kw. |
| #15 | (space adj2 change*).ti,ab,kw. |
| #16 | migrat*.ti,ab,kw. |
| #17 | drift.ti,ab,kw. |
| #18 | tooth migration/exp |
| #19 | #13 OR #14 OR #15 OR #16 OR #17 OR #18 |
| #20 | #6 AND #12 AND #19 |
