# Supplementary material for: Space changes after premature loss of mandibular second primary molars: a systematic review and meta-analysis
Source: BMC Oral Health. 2026 Oct 30;26:2149. doi: 10.1186/s12903-026-10044-8 (PMC13721352; doi:10.1186/s12903-026-10044-8)
Supplement: Supplementary file 3 [file 12903_2026_10044_MOESM3_ESM.docx]

**Supplementary Table S1: Detailed numerical outcomes per study**

| **Author** | **Study Design** | **Setting / Country** | **Age (y)** | **Sample Size (n)** | **Arch (n)** | **Groups** | **Data Collection** | **Follow-up** | **Evaluation Indicators** | **E-Space Loss Mean ± SD (mm)** | **Arch Length Change Mean ± SD (mm)** | **Arch Hemi-Perimeter Change Mean ± SD (mm)** | **Other Outcomes** |
| --- | --- | --- | --- | --- | --- | --- | --- | --- | --- | --- | --- | --- | --- |
| **Macena et al., 2011** | Longitudinal observational study with a split-mouth design | Brazil | 6–9 y | 55ᵃ | Maxilla (13) Mandible (22) (2nd molar cases only) | **2 groups:** Case: extraction of a primary molar Control: no extraction of contralateral molar | Clinical examination, cast analysis, and radiographic analysis | 3, 6, 10 months | Dental space at extraction site, arch length, arch hemi-perimeter | **Case group:** **Maxilla:**  **Initial–3M:** 1.2 ± 0.8 mm  **3–6M:** −0.1 ± 0.5 mm  **6–10M:** −0.4 ± 0.5 mm **Mandible:**  **Initial–3M:** 0.8 ± 0.7 mm  **3–6M:** 0.3 ± 0.4 mm  **6–10M:** 0.1 ± 0.3 mm **Control group:** Not reported | **Case group:** **Maxilla:**  **Initial–3M:** 0.4 ± 0.5 mm  **3–6M:** 0.0 ± 0.3 mm  **6–10M:** −0.2 ± 0.4 mm **Mandible:**  **Initial–3M:** 0.2 ± 0.5 mm  **3–6M:** 0.1 ± 0.4 mm  **6–10M:** 0.0 ± 0.4 mm | **Case group:** **Maxilla:**  **Initial–3M:** 0.2 ± 0.7 mm  **3–6M:** 0.0 ± 0.7 mm  **6–10M:** −0.1 ± 0.7 mm **Mandible:**  **Initial–3M:** 0.6 ± 0.6 mm  **3–6M:** 0.5 ± 0.8 mm  **6–10M:** 0.0 ± 0.6 mm **Control group:** **Maxilla:**  **Initial–3M:** 0.1 ± 0.5 mm  **3–6M:** −0.1 ± 0.7 mm  **6–10M:** −0.3 ± 0.5 mm **Mandible:**  **Initial–3M:** 0.1 ± 0.6 mm  **3–6M:** 0.1 ± 0.7 mm  **6–10M:** 0.0 ± 1.0 mm | Measurement of space loss, arch length and hemi-perimeter changes before and after extraction of second primary molars |
| **Bindayel, 2019** | Longitudinal observational study with a split-mouth design | Saudi Arabia | 7.5–10.7 (Mean: 9 ± 1.5) | 32 | Maxilla (12) Mandible (20) | **2 groups:** Case: extraction of a primary molar Control: no extraction of contralateral molar | Dental casts and radiographs | 3 weeks | Dental space at extraction and control sites; developmental stage of the premolar | **Case group:**  **Maxilla:** 0.45 ± 0.45 mm  **Mandible:** 0.49 ± 0.33 mm **Control group:** Not reported | N/A | N/A | **Angle's molar relationship:** Mean space loss was higher with Class II vs Class I molar relationship (P = 0.234; NS).  **Developmental stage of premolar:** Stage of premolar development did not significantly correlate with space loss (P = 0.811). |
| **Kundra et al., 2024** | Longitudinal observational study with a split-mouth design | India | 6–9 (Mean: 6.95 ± 0.83) | 20ᵇ | Maxilla (8) Mandible (12) (2nd molar cases only: n=6) | **2 groups:** Case: extraction of a primary molar Control: no extraction of contralateral molar | Clinical examination, radiological analysis, and 3D CT digital images of plaster casts | 9 months | Dental space at extraction and control sides; angulation of first permanent molar; arch length, arch width, hemi-perimeter | **Maxilla:**  **Case group:** 1.1 ± 0.57 mm  **Control group:** 0 mm (no change) **Mandible:**  **Case group:** 1.57 ± 0.15 mm  **Control group:** 0 mm (no change) | **Before extraction:** 22.39 ± 3.05 mm **After 9 months:** 20.58 ± 3.50 mm | **Before extraction:** 28.06 ± 2.47 mm **After 9 months:** 25.26 ± 2.80 mm | **Angulation of first permanent molar:** Case group: 2.28° difference in maxilla (P = 0.056); 3.74° difference in mandible (P = 0.003) Control group: No change after 9 months |
| **de Souza et al., 2024** | Two-arm randomised clinical trial | Brazil | 6–9 (Mean: 7.45 ± 0.92) | 26ᶜ IG: 12 CG: 14 | Mandible (26) IG (12) / CG (14) (CG 2nd molar: n=10) | **2 groups:** IG: lower primary molar lost + band-and-loop space maintainer placed CG: lower primary molar lost, no space maintainer | Digital calipers on dental casts | 3, 6 months | Space changes, arch perimeter, intercanine length and width, arch length, arch width | **IG (n=13):**  **Initial–3M:** −0.3 ± 0.50 mm  **3–6M:** −0.3 ± 0.45 mm **CG (n=13):**  **Initial–3M:** −0.8 ± 0.44 mm  **3–6M:** −0.3 ± 0.44 mm | **IG:**  **Initial–3M:** −0.2 (SD 0.9) mm  **3–6M:** 0.0 (SD 0.35) mm **CG:**  **Initial–3M:** −0.1 (SD 0.97) mm  **3–6M:** −0.6 (SD 0.85) mm | **IG:**  **Initial–3M:** −0.2 (SD 1.51) mm  **3–6M:** −0.4 (SD 1.03) mm **CG:**  **Initial–3M:** −1.1 (SD 0.9) mm  **3–6M:** 0.2 (SD 1.09) mm | **Changes in arch width:** IG: Initial–3M: −0.1 (0.73); 3–6M: −0.5 (1.02) mm CG: Initial–3M: −0.6 (0.84); 3–6M: 0.3 (0.88) mm  **Intercanine length:** IG: Initial–3M: 0.2 (0.46); 3–6M: 0.1 (0.42) mm CG: Initial–3M: 0.3 (0.69); 3–6M: 0.0 (0.29) mm  **Intercuspation of first permanent molars:** Space loss in CG without intercuspation: −2.0 (0.71) mm vs. −0.7 (0.27) mm with intercuspation (P = 0.007) |

**Notes:** IG = Intervention Group; CG = Control Group; M = months; NS = not significant; N/A = not applicable.
**ᵃ** Macena et al. (2011): total sample size was 55 children; only second molar cases (n=35, of which mandibular n=22) were included in the meta-analysis.
**ᵇ** Kundra et al. (2024): total sample size was 20 children; only mandibular second molar cases (n=6) were included in the meta-analysis.
**ᶜ** de Souza et al. (2024): IG n=12, CG n=14 (total 26); only the control arm (n=14) was considered, of which 10 had second primary molar loss and were included in the meta-analysis.
